# Mitochondrial NADH dehydrogenase polymorphisms are associated with breast cancer in Poland

**DOI:** 10.1007/s13353-013-0190-9

**Published:** 2014-01-11

**Authors:** Ludmiła Grzybowska-Szatkowska, Brygida Ślaska

**Affiliations:** 1 Department of Oncology, Medical University of Lublin, Jaczewskiego 7, 20-090 Lublin, Poland; 2 St. John’s Cancer Centre, The Regional Oncology Centre of Lublin, Jaczewskiego 7, 20-090 Lublin, Poland; 3 Department of Biological Bases of Animal Production, University of Life Sciences in Lublin, Akademicka 13, 20-950 Lublin, Poland

**Keywords:** Cancer, Carcinogenesis, Mitochondrial DNA mutation

## Abstract

Complex I NADH-oxidoreductase-ubiquinone transports reducing equivalents from the reduced form of NADH to ubiquinone (coenzyme Q-CoQ). The purpose of this study was to analyze mutations in *MT*-*ND1*, *MT*-*ND2*, *MT*-*ND3* and *MT*-*ND6* genes and their effect on the biochemical properties, structure and functioning of proteins in patients with breast tumours. In research materials, in 50 patients, 28 total polymorphisms and five mutations were detected. Most detected polymorphisms (50 %, 14/28) were observed in *MT*-*ND2* gene. Most of them were silent mutations. Five polymorphisms (m.G3916A, m.C4888T, m.A4918G, m.C5363T, m.C10283T) do not exist in the database. A total of five mutations in 13 patients (13/50) were detected, including two not described in the literature: m.C4987G and m.T10173C. It cannot be excluded that, through the mutations and polymorphism impact on the protein structure, they may cause mitochondrial dysfunction and contribute to the appearance of other changes in mtDNA. The results of our study indicate the presence of homological changes in the sequence of mtDNA in both breast cancer and in some mitochondrial diseases. Mutations in the examined genes in breast cancer may affect the cell and cause its dysfunction, as is the case in mitochondrial diseases.

## Introduction

The participation of mitochondria in tumorigenesis was first suggested by Warburg in 1932. His explanation was prevalence of anaerobic glycolysis in cancer cells. Mitochondrial DNA is composed of base pairs 16569 (Andrews et al. 1999). Almost every nucleotide encodes genetic information. The mtDNA encodes genes for two types of rRNA, 22 types of tRNA and 13 proteins. Two chains of mtDNA, both light and heavy, are coding chains. Most of the genes are located in the heavy chain. The encoded light chain genes are merely the eight types of tRNA genes and one of the complex I of the respiratory chain (*MT*-*ND6*). The heavy chain of other genes encodes complex I (*MT*-*ND1*, *MT*-*ND2*, *MT*-*ND3*, *MT*-*ND4*, *MT*-*ND4L* and *MT*-*ND5*) and the rest of the 13 proteins are involved in the mitochondrial oxidative phosphorylation (OXPHOS). In mtDNA the first mutation associated with cancer was described in renal cell carcinoma (RCC). It concerned the *MT*-*ND1* gene for oxidative phosphorylation (Welter et al. 1989). The importance of changes taking place in the mitochondria in the process of carcinogenesis suggests linking mev −1 mutations in a gene *SDHC* in *Caenorhabditis elegans* are connected with an increased production of reactive oxygen species (ROS), which are very important factors in the inactivation of proteins associated with apoptosis and neoplasia, such as p 16INK4a and p53 (Grzybowska - Szatkowska and Slaska 2012a). The purpose of this study was to analyze mutations in *MT*-*ND1*, *MT*-*ND2*, *MT*-*ND3* and *MT*-*ND6* genes and their effect on the biochemical properties, structure and functioning of proteins in patients with breast tumours.

## Material and methods

The tested material was DNA isolated from specimens of ductal carcinoma (*carcinoma ductale*) Tp1-2 Np0-1Mp0 and blood sampled from 50 patients who had also undergone surgery for breast cancer. The patients had received no chemo- or hormonal therapy. They were all perimenopausal. The blood was taken from each patient and placed in sterile vacuum test tubes containing dipotassium ethylenediaminetetraacetic acid anti-coagulant. The DNA was isolated with the use of an automated nucleic acid extraction system-QIACube (Qiagen, Hilden, Germany). It was extracted from whole peripheral blood with the QIAamp DNA Blood Mini Kit (Qiagen), and the QIAamp DNA Blood Kit (Qiagen) was used for DNA extraction from the tissue. Sequences of *MT*-*ND1*, *MT*-*ND2*, *MT*-*ND3*, and *MT*-*ND6* genes were analysed (Table 1). Based on the *Homo sapiens* mitochondrion, the complete genome (AC_000021) sequence, primers for the fragments of test genes (Table 1) were designed with the use of the Primer3 programme (http://frodo.wi.mit.edu/). The amplification products were visualised in 2 % agarose gel. Both strands of the particular genes were sequenced. Amplicons were sequenced using the BigDye Terminator Cycle Sequencing Kit (Applied Biosystem, Foster City, CA, USA) in the GeneAmp PCR system 9700 (Applied Biosystem). The samples were subsequently purified on CentriSep columns according to the manufacturer’s protocol or precipitated with ethanol and sodium acetate according to the protocol of the BigDye Kit manufacturer. The extension products were separated on the ABI 377 automated sequencer (Applied Biosystem). After establishing the consensus sequence for each analysed sequence, the sequences of the genes were compared with the Cambridge reference sequence of the human mitochondrial DNA (AC_000021). The single nucleotide polymorphisms established for particular gene fragments were used to identify haplotypes. Polymorphisms were considered to be changes that occur in both blood/normal breast cell and tumour cells in the same patient. Mutation is a change characteristic only for cancer cells and not occurring in a patient’s blood/normal breast cell.

**Table 1:** Primer sequences used in PCR amplification and sequencing

Gene	Gene name	Gene position in mtDNA (bp) AC_000021	Primer position (bp)	Primer sequence 5′-3′
*MT*-*ND1*	Mitochondrially encoded NADH dehydrogenase 1	3307..4262	3170..3190	CCCGTAAATGATATCATCTCA
			3569..3488	AGGGGCTCTTTGGTGAAGAG
			3868.. 3889	TCCACACTAGCAGAGACCAAC
			3520..3539	ATCACCGCCCCGACCTTAG
			4517..4539	GCTTAGCGCTGTGATGAGTGTG
*MT*-*ND2*	Mitochondrially encoded NADH dehydrogenase 2	4470..5511	5049.. 5074	CTACCGTACAACCCTAACATAACCA
			5420..5439	GGGTGGGTTTTGTATGTTCA
			4885..4911	GGGAGAGATTTGGTATATGATTGAGA
			5531..5576	AATTAAGTATTGCAACTTACTGAGG
			4394..4416	CATCCTAAAGTAAGGTCAGCTA
*MT*-*ND3*	Mitochondrially encoded NADH dehydrogenase 3	10059..10404	9917..9935	CGCCGCCTGATACTGGCAT
			10511..10530	CTAGTATTCCTAGAAGTGAG
*MT*-*ND6*	Mitochondrially encoded NADH dehydrogenase 6	complement (14149..14673)	14051..14073	CCACCTCCATCATCACCTCAAC
			14716..14743	GTTCTTGTAGTTGAAATACAACGATGG

The impact on the physical and biochemical peptide properties. The probability of deleterious mutations, i.e. the functional effect of the non-synonymous (amino acid-changing) protein coding SNP, was determined using the Panther Classification System (http://www.pantherdb.org/), which estimates the value of substitution position-specific evolutionary conservation (subPSEC) and the probability of a deleterious effect on protein function (P_deleterious_, Probability of Functional Impairment) on the basis of the alignment of evolutionarily determined proteins. PANTHER subPSEC scores are continuous values from 0 (neutral) to about −10 (most likely to be deleterious); -3 is the previously identified cutoff point for functional significance. A cutoff of −3 corresponds to a 50 % probability that a score is deleterious. From this, the probability that a given variant will cause a deleterious effect on protein function is estimated by P_deleterious_, such that a subPSEC score of −3 corresponds to a P_deleterious_ of 0.5. The determination of helicity per residue for the peptides was performed using the AGADIR program (http://agadir.crg.es/). The GRAVY and the theoretical pI were obtained using the software program ProtParam tool (http://web.expasy.org/). The incidence of amino acid in each position of a protein sequence was assessed using position specific scoring matrix (PSSM). PSSM scores are generally shown as positive or negative integers. Positive scores indicate that a given amino acid substitution occurs more frequently in an alignment than expected by chance, while negative scores indicate that a substitution occurs less frequently than expected. Large positive scores often indicate critical functional residues, which may signify active site residues or residues required for other intermolecular interactions (http://www.ncbi.nlm.nih.gov/Class/Structure/pssm/pssm_viewer.cgi).

The Institutional Review Board at the Medical University of Lublin specifically approved this study. Approval Number KE-254/141/2009.

## Results

The results are shown in Tables 2, 3, 4 and 5. In the study, 50 patients had a total number of 28 polymorphisms and five mutations (Table 2). Most polymorphisms (50 %, 14/28) occurred in the *MT*-*ND2* gene sequence. Most of them were silent mutations. Five polymorphisms (m.G3916A, m.C4888T, m.A4918G, m.C5363T, m.C10283T) did not exist in the database. Polymorphisms m.G3916A, m.C4888T, m.A4918G, m.C10283T are of the missense type and in the case of two of them Pdt was above 0.5 which indicates that those changes are not indifferent to the function of the protein (Table 4). A total of five mutations were detected in 13 patients (13/50), including two not described in the literature (m.C4987G, m.T10173C). Only a change of p.C39R in *MT*-*ND3* seems to have an impact on the functioning of the protein, Pdt subPSEC = 0.86366 -4.84604 (Table 4). The amino acid of cysteine containing sulfhydryl group, which is also very uncommon in proteins, was replaced here with arginine containing the guanidine group. This mutation converts a highly conserved cysteine (PSSM score = 11) to argnine (PSSM score = −5) (Table 5).

**Table 2:** Differences in *MT*-*ND1*, *MT*-*ND2*, *MT*-*ND3* and *MT*-*ND6* genes sequences between reference Cambridge sequence and examined material

Number of patients (patient’s no)	Frequency (mtDB)					Mitochondrial haplogroup: according to van Oven and Kayser (2009)	Cambridge reference sequences	Sequences in breast cancer cell	Sequences in patient’s blood	Sequences in normal breast cell	Aminoacid change
	A	G	C	T	del						
*MT*-*ND1* polymorphism											
1(1)	20	2684	–	–	–	U6a1h,U1a1,U6a5,H9a,I2e, M27b,M38b,C5a,G2b2c	m.G3591	m.G3591A	m.G3591A	m.G3591A	synonim
1(213)	–	–	2698	6	–	L0a2c,P10,U8a1	m.C3738	m.C3738T	m.C3738T	m.C3738T	synonim
1(5)	11	2692	–	–	–	K1b1a2,U9,HV4c,H2a1c, T2c1c1,T2g1, T2g2,N-Y1, D4a3b2, L0a2a1b	m.G3834	m.G3834A	m.G3834A	m.G3834A	synonim
3(19,20,21)	–	–	–	–	–	–	m.G3916	m.G3916A^a^	m.G3916A^a^	m.G3916A^a^	p.E204K
1(20)			2680	24		H3g1,H4,T2b4c,L3k	m.C3992	m.C3992T	m.C3992T	m.C3992T	p.T229M
4(15,32,82,213)	–	–	244	2460	–	L1b1a3b,L4b1,M2a1b,M2c,	m.T4216	m.T4216C	m.T4216C	m.T4216C	p.Y304H
						M14,D5c,X2b7,R2′JT,P4a,					
						K1a2c,H1bm,H10a					
1(217)	–	–	2703	1	–	L0,L3c,V15,H1n6,H73	m.C4221	m.C4221A	m.C4221A	m.C4221A	synonim
*MT*-*ND1* mutation											
1(31)	2677	10	–	17	–	L1c1,M21a,B2f,H1b1	m.A3796	m.A3796G	m.A3796A	m.A3796A	p.T164A
*MT*-*ND2* polymorphism											
1(3,212)	2669	2		33	–	N1ab1	m.A4529	m.A4529T	m.A4529T	m.A4529T	synonim
3(4,26)	77	2627	–	–	–	M3a,V	m.G4580	m.G4580A	m.G4580A	m.G4580A	synonym
1(4)	–	–	22	2682	–	M22 V1a,H1a1,H13a2b1	m.T4639	m.T4639C	m.T4639C	m.T4639C	p.I57T
47(1–19,22-40, 202,203,212,213,217,82,83,84,81	2695	96	–	–	–	H6a1,H13a1b	m.A4727	m.A4727G	m.A4727G	m.A4727G	synonym
27(1–2,4,6-11,15-16,18,20-23,27-35,203, 212, 213, 217,219)	30	2674	–	–	–	R2,J2b1d,,F2e, B4a1a1a3, H2a,H2a1d,	m.A4769	m.A4769G	m.A4769G	m.AG4769G	synonim
1(32)	–	–	12	2692	–	L2a5,L3e2a,T2c1c,_,_B4a2a, U5a1d2a1,K1a1b1f	m.T4823	m.T4823 C	m.T4823 C	m.T4823C	synonym
1(6)	1	2703	–	–	–	Z7	m.G4841	m.G4841A	m.G4841A	m.G4841A	synonym
1(26)	–	–	–	–	–	–	m.C4888	m.C4888T^a^	m.C4888T^a^	m.C4888T^a^	p.S140L
4(13,15,87,32)	–	–	–	–	–	–	m.A4918	m.A4918G^a^	m.A4918G^a^	m.A4918G^a^	p.N150S
1(1)	15	2689	–	–	–	M63,D2a2,J1b6a,T1a,U1a	m.G4991	m.G4991A	m.G4991A	m.G4991A	synonim
3(27,32,213,)	2569	135	–	–	–	L2a4,L0a2,L5a,L2a1,L3d, M30b,M5b1,M5c2,M75, N9b,Y2,T2b,F1b1,HV2a,H3b1,U1c,U2d2,U5b3g,U6d1a	m.G5147	m.G5147A	m.G5147A	m.G5147A	synonim
1(35)	2698	6	–	–	–	U8a1	m.A5240	m.A5240G	m.A5240G	m.A5240G	synonim
1(4)	–	–	–	–	–		m.C5363	m.C5363T^a^	m.C5363T^a^	m.C5363T^a^	synonim
1(23)	176	2528	–	–	–	K1a12, U2d2, H1e,H2a2b4, H6a1a1, H41a,J1b1,J2b2, B2k, B4e,P4,P4b1,W, A2af, A2a1b2,O1a,M7b′d, M52′58,M7f,C4a1e,G4, Q4,Q1′2, Q2b,Q3b,,L3e1c, L3h2,L0a,L0d3,L1c1d, L4,L4b2	m.G5460	m.G5460A	m.G5460A	m.G5460A	p.A331T
*MT*-*ND2* mutation											
1(30)	–	–	–	–	–		m.G4580	m.G4580A^a^	m.G4580G	m.G4580G	synonim
10(6,7,20,26,27, 34,82,203,217, 219)	–	–	–	–	–		m.C4987	m.C4987G^a^	m.CC4987	m.CC4987	p.T173S
*MT*-*ND3* polymorphism											
1(203)	– 1461	– 1242	82 –	2621 –	– –	N1,B4a1, R0a2k1,R12′21,P4,J,J1c8, R11, K1, B4c1,B5 N, N1a1, N8, Y,S3, L3e1a, L1c1a1	m.T10238 m.A10398	m.T10238C m.A10398G	m.T10238C m.A10398G	m.T10238C m.A10398G	synonim p.T114A
1(32)							m.C10281	m.C10281T	m.C10281T	m.C10281T	p.L75V
1(17)	2699	5	–	–	–	Q2b,F1a1c1, HV11a,H59a, U5b1e	m.A10283	m.A10283G^a^	m.A10283G^a^	m.A10283G^a^	synonim
2(23,212)	1461	1242	–	–	–	R0a2k1,R12′21,P4,J,J1c8, R11, K1, B4c1,B5 N, N1a1, N8, Y,S3, L3e1a, L1c1a1	m.A10398	m.A10398G	m.A10398G	m.A10398G	p.T114A
*MT*-*ND3* mutation											
1(213)	–	–	–	–	–		m.T10173	m.T10173C^a^	m.T10173	m.T10173	p.C39R
*MT*-*ND6* polymorphism											
1(35)	– 2700	– –	2698 –	6 4	– –	B5a2a,,H44a M7a1a4,R2c,H6c1	m.C14149 m.A14185	m.C14149A^a^ m.A4185C	m.C14149A m.A14185C	m.C14149A m.A14185C	synonim synonim
1(27)	2700 2613	– 91	– –	4 –	– –	M7a1a4,R2c,H6c1 I5,T2,T5,M72,	m.A14185 m.A14233	m.A14185C m.A14233G	m.A14185C m.A14233G	m.A14185C m.A14233G	synonim synonim
2(26,32,213)	2613	91	–	–	–	I5,T2,T5,M72	m.A14233	m.A14233G	m.A14233G	m.A14233G	synonim
1(1)	58	2646	–	–	–	L012,M7a1,D1b,N3Ab,A2c, H18,U1a′c,U6a3a1	m.G14364	m.G14364A	m.G14364A	m.G14364A	synonim
1(2)	2700	4	–	–	–	M3c1b,M18b,N22a,X1,B4b1c, HV1a2a,H11a2, L3c, L3h1a2a	m.A14587	m.A14587G	m.A14587G	m.A14587G	synonim
*MT*-*ND6* mutation											
1(35)	–	–	–	–	–	–	m.T14166	m.T14166C	m.T14166	m.T14166	p.I170V

^a^Positions in which mutations or polymorphisms were described in literature for the first time

**Table 3:** Comparison of protein properties in non-synonymous protein-coding SNP in females with breast cancer

Amino acid change	Theoretical pI (Isoelectric point)	Aliphatic index	Instability index	Grand average of hydrophobicity (GRAVY)	Percentage helix (H) (start and end of helix)
*MT*-*ND1*					
p.T164A	6.11	123.40	41.94	0.690	H4 = 0.49 (146–166)
p.E204K	7.85	123.08	41.31	0.681	Mitochondrial matrix
p.T229M	6.11	123.08	44.06	0.690	Mitochondrial matrix
p.Y304K	6.29	123.08	42.69	0.676	H8 = 3.71 (294–314)
Normal	6.11	123.08	41.94	0.682	H4 = 0.48
					H8 = 6.59
*MT*-*ND2*					
p.I57T	9.84	118.10	34.59	0.621	H2 = 2.91
p.S140L	9.84	120.06	34.10	0.651	H4 = 0.97 (123–143)
p.N150S	9.84	119.22	34.35	0.644	H5 = 3.74 (149–169)
p.T173S	9.84	119.22	36.38	0.636	Mitochondrial matrix
p.A331T	9.84	118.93	34.35	0.629	H10 = 0.37 (326–345)
Normal	9.84	119.22	34.35	0.636	H2 = 12.89
					H4 = 0.95
					H5 = 3.92
					H10 = 0.30
*MT*-*ND3*					
p.C39R	4.56	46.06	140.00	0.931	Mitochondrial matrix
p.L75V	4.33	52.30	139.13	0.996	H2 = 4.19 (55–75)
p.T114A	4.33	48.95	140.87	1.014	Mitochondrial matrix
Normal	4.33	50.62	140.00	0.992	H2 = 4.20
*MT*-*ND6*					
p.I170V	4.18	28.38	125.17	1.069	H6 = 0.23 (151–171)
Normal	4.18	29.48	125.75	1.071	N6 = 0.23

**Table 4:** Probability of a functional effect on the non-synonymous protein-coding SNP

subPSEC	P_deleterious_	Substitution	MSA position	P_wt_	P_substituted_	NIC
*MT*-*ND1*						
−1.55746	0.19115	p.T164A	136	0.11421	0.06006	2.788
−2.97215	0.49304	p.E204K	176	0.46202	0.04771	2.729
−1.76426	0.22518	p.T229M	201	0.0994	0.04119	2.768
−0.92687	0.11174	p.Y304H	276	0.10488	0.06968	1.777
*MT*-*ND2*						
−2.29998	0.33181	p.I57T	52	0.26164	0.1134	5.16
−3.70833	0.67003	p.S140L	129	0.11788	0.02633	12.218
−3.06754	0.51688	p.N150S	140	0.11018	0.04529	11.003
−2.82675	0.4568	p.T173S	0.81226	0.00678	1.863	0.81226
−2.46689	0.36979	p.A331T	344	0.11939	0.14735	11.076
*MT*-*ND3*						
−4.84604	0.86366	p.C39R	0.81226	0.00678	1.863	0.81226
−2.64329	0.41176	p.L75V	71	0.59287	0.05843	1.845
−0.64843	0.08694	p.T114A	110	0.24587	0.29884	1.636
*MT*-*ND6*						
−0.88247	0.1074	p.I170V	0.36272	0.27109	1.92	0.36272

**Table 5:** Residue frequencies and PSSM score determined by using PSSM viewer program

Residue	Raw frequency	Weighted frequency	PSSM score
*MT*-*ND1*-MTH00104			
p.T164A			
T	0.77	0.63	5
A	0.05	0.08	0
p.E204K			
E	1.00	1.00	7
K	–	–	0
p.T229M			
T	0.25	0.27	4
M	0.11	0.17	4
p.H304Y			
H	0.69	0.54	8
Y	0.24	0.32	6
*MT*-*ND2*-MTH00105			
p.I57T			
T	0.79	0.73	7
I	0.17	0.23	3
p.S140L			
S	0.92	0.87	6
L	0.01	0.01	−3
p.N150S			
N	0.94	0.90	8
S	0.00	0.01	−1
p.T173S			
T	0.98	0.95	7
S	0.00	0.01	0
p.A331T			
T	0.10	0.13	2
A	0.01	0.01	−3
*MT*-*ND3*-MTH00106			
p.C39R			
C	1.00	0.99	11
R			−5
p.L75V			
L	0.99	0.93	6
V			−1
p.T114A			
T	0.60	0.55	5
A	0.18	0.15	1
*MT*-*ND1*-MTH00109			
p.I170V			
V	0.71	0.52	5
I	0.27	0.44	6

## Discussion

Mitochondrial electron transport chain consists of four large complexes of respiratory enzymes. Mutations in the OXPHOS genes in mtDNA cause disturbances in the transport of electrons through the respiratory chain. Complex I is called oxidoreductases-NADH-ubiquinone. It consists of several flavine mononucelotide polypeptides and iron-sulfur proteins. The purpose of the complex is to transport reducing equivalents from the reduced form of NADH to ubiquinone (coenzyme Q-CoQ). Mutations in the genes in mtDNA OXPHOS cause disturbances in the transport of electrons through the respiratory chain leading to optic nerve death and complete blindness (Brown et al. 1992). The severity of the symptoms depend on the scope and location of the mutation (Komaki et al. 2003; Mitchell et al. 2006; Grzybowska-Szatkowska and Slaska 2012a).

Although most of the pathogenic mutations in mitochondrial diseases are described as homoplasmic, some are also heteroplasmic. Most occur in LHON (Leber ’s hereditary optic neuropathy), Kearns - Sayre syndrome (Moraes et al. 1989; Brown et al. 1992) and in non-insulin dependent diabetes (Perucca - Lostanlen et al. 2002). They are also described in canine tumours (Slaska et al. 2013a, b). Tan et al. (2002) reported in breast cancer four somatic changes in the OXPHOS system. In three cases they were related to *MT*-*ND2* (silent mutations). The change in the position m.T3398C of *MT*-*ND1* described in breast cancer is also observed in patients with progressive external ophtalmoplegy and cardiomyopathy (Jaksch et al. 1996; Carelli et al. 2004). On the other hand, Bai et al. (2000) have found a reduced expression of mRNA for this complex in the case of mutations in *MT*-*ND5* complex I in murine lung cancer cells. The authors believe that it might be caused by disturbances of the regulation of transcription (up- regulation), mRNA stability or impairment of selective degradation of the mutant mRNA.

In breast cancer carcinoma, sequence analysis of all genes encoding mt-tRNA revealed eight polymorphisms and two mutations detected in 34 % of the patients (Grzybowska-Szatkowska and Slaska 2012b). Transitions m.A15924G and m.A12308G took place only in neoplastic cells, but not in the blood, so the mutations can be attributed strictly to a neoplastic process. The authors suggest that it may be a result of the secondary and tertiary tRNA structure and that the polymorphisms may lead to mitochondrial dysfunction and contribute to revealing other changes in mtDNA (Grzybowska-Szatkowska and Slaska 2012b). Two other polymorphisms m.G10398A and m.T10400C are also connected with a high risk of breast cancer (Mims et al. 2005; Czarnecka et al. 2010; Sultana et al. 2011).

The m.G10398A polymorphism is a marker of haplogroup IJK. Setiawan et al. (2008) deny the link between the m.G10398A polymorphism and breast cancer. Also, the polymorphism of *MT*-*ND2*: m.A4769G and m.G5460A are found in breast cancer (Czarnecka et al. 2010). In this paper, the m.G10398A polymorphism was observed in three patients in the material while m.A4769G was present in 27 cases, and m.G5460A only in one patient (Table 2). The polymorphism m.A4769G in contrast to the other two is synonymous, and does not change the amino acid into the protein. Both the transition m.G10398A (resulting in the amino acid change p.T114A) and m.G5460A (resulting in the amino acid change p.A331T) are not present in a high conservation region and, by Panther Pdt, it is less than 0.5 (Table 4).

Replacement of polar threonine with alanine causes a reduction in the non-polar aliphatic index of the protein and the amino acid alanine is less preferred in that position than the threonine (Table 5). The m.G5460A polymorphism is described in Alzheimer’s disease (AD), although the relationship between AD and the polymorphism is being discussed (Mitchell et al. 2006). The test material (Table 2) also detected two polymorphisms described in LHON (Johns et al. 1991; Fauser et al. 2002) and adult onset dystonia. Mitochondrial haplogroup J/T (Herrnstadt et al. 2002) is defined by the transition at position m.T4216C. This polymorphism has been described as a secondary mutation in LHON (Carelli et al. 2004) and also to be associated with insulin resistance and type 2 diabetes (Crispim et al. 2006).

This polymorphism relates to helix 8 and changes its percentage of 6.59 to 3.71 (Table 3). In insulin resistance and type 2 diabetes it often occurs together with transitions in *MT*-*ND2* at position 4917 (Johns et al. 1991). In the material studied by us, there was no transition at position 4917, but at position m.A4918G (Table 2), which resulted in an amino acid change in the same codon (p.N150S) (Johns et al. 1991; Crispim et al. 2006). This transition was accompanied by a polymorphism in m.T4216C in two patients (Table 2).

Transition m.A4918T is described in the literature as a natural variant of mtDNA (Marzuki et al. 1991) and it converts a highly conserved arginine containing an amide group to the serine with a hydroxyl group. Also, the evolutionary analysis of coding SNPs subPSEC was below −3, indicating that the polymorphism has an impact on the functioning of the protein (Table 4). The polymorphism m.T4639C refers to a highly conserved region and occurs in haplogrup M22 (Table 2), and also relates to the codon described in LHON.

In LHON, polymorphism in that codon concerns mtDNA position 4640 and there is a replacement of isoleucine with methionine there (p.I57M) (Volod’ko et al. 2006; Pereira et al. 2011). It should be noted that the substitution of isoleucine for thereonine will change the percentage of the second helix down to 2.91 from 12.89 (Table 3). Transition at mtDNA position 4888 on the *MT*-*ND2* occurred in both tumour cells and blood so it should be taken as a polymorphism (Table 2). The polymorphism has not been described so far in the literature and is not present in the database. It causes a conversion of polar leucine to nonpolar serine and the evolutionary analysis of coding SNPs subPSEC was less than −3 (Table 4), which indicates that the polymorphism is not indifferent to the functioning of the protein.

The polymorphism m.C3992T is relatively rare in the general population and when associated with the mitochondrial haplogrups of H, T, L (Table 2) it appeared as a mutation in thyroid cancer (Maximo et al. 2002; Brandon et al. 2006). We currently know mtDNA genes and regions in which polymorphisms and mutations are associated with similar types of cancer in the corresponding tissues of humans and dogs (Ślaska et al. 2013). In the different types of tumours in dogs, including breastcancer, there are a number of polymorphisms described in the D-loop region, *MT*-*CYTB*, *MT*-*COI* and *MT*-*ND1* gene, and a missense mutation in *epithelioma glandulae sebacei* resulting in the amino acid change p.T193N leading to a change in *MT*-*ND1* (Slaska et al. 2013a, b). Heteroplasmic changes were found in *MT*-*ND1* and *MT*-*CYTB* in *epithelioma glandulae sebacei* and in *MT*-*CYTB* in *lymphoma centroblasticum* (Slaska et al. 2013).

Out of the five detected, two mutations were not reported in the literature. The m.A3796G mutation (resulting in the amino acid change p.T164A), which is present in the literature, was described in adult onset dystonia (Simon et al. 2003; Mitchell et al. 2006). The m.G4580A silent mutation was described in pancreatic cancer cells (Jones et al. 2001). Both the mutation m.C4987G (resulting in the amino acid change p.T173S) and the m.T10173C (resulting in the amino acid change p.C39R) concern highly conserved aminoacids. The m.C4987G mutation occurred in up to ten patients (Table 2). The literature describes the mutation in the codon 173. Those were related to position 4986. That mutation caused replacement of threonine with alanine. In the cells of intestinal crypts (Taylor et al. 2003) and in the case of oral cancer – by proline (Tan et al. 2004).

The mutation m.T14166C (resulting in the amino acid change p.I70V) was described in neurogastrointestinal mitochondrial encephalomyopathy (MNGIE) (Nishigaki et al. 2003). MNGIE is an autosomal recessive disorder caused by loss-of-function mutations in the gene encoding thymidine phosphorylase. All changes detected in our study were homplasmic (Table 2). In case of changes in tumours we often have to deal with homoplasia. As a result of intramitochondial selection, the dominance of one type of mtDNA occurs in a mitochondrion, a so-called functional advantage (Augenlicht and Heerdt 2001; Jones et al. 2001; Grzybowska-Szatkowska and Slaska 2012a). During cell division the cell strives to obtain a prevalence of one type of mRNA (a homoplasmy - a replicative segregation) (Augenlicht and Heerdt 2001; Jones et al. 2001; Grzybowska-Szatkowska and Slaska 2012a).

The period required for the replication of segregation to occur would correspond with the phase of neoplastic transformation. The homoplasmy can also occur as a result of a random segregation of mitochondria during cell division (Coller et al. 2001). In subsequent generations of progenitor cells heteroplasmy may persist, or by a genetic drift a homoplasmy may occur (Coller et al. 2001; Jones et al. 2001).

As a result of genetic drift either elimination or stabilisation of rare variants of mtDNA occurs. It seems that this may be the cause of carcinogenesis. The occurrence and severity of symptoms in mitochondrial diseases depends on the ratio between the normal and mutated DNA. The dominance of mutant mtDNA in a cell leads to disturbances in the production of energy in the process of oxidative phosphorylation, which may lead to cell and tissue damage. The clinical manifestations of those disorders depends on the type of defects in the mitochondrial DNA and on the degree of sensitivity of the heteroplasmic tissue disorders related to cellular respiration. When there is an advantage of mutant mtDNA, symptoms arise that are progressive in character.

Mutations in the genes for mitochondrial proteins cause a wide range of symptoms. Changes in mtDNA accumulate with age due to long-term exposure to free radicals generated in the mitochondria (Liu et al. 1998; Czarnecka and Bartnik 2011). The delayed onset and progressive nature of mitochondrial diseases indicate a progressive deterioration with age in the mitochondrial function (Liu et al. 1998). This is why some mitochondrial diseases occur in middle or old age (Brown et al. 1992; Lyamzaev et al. 2004).

In the studied material we detected polymorphisms occurring in patients with LHON syndrome and in adult onset dystonia. In the phase of clinical detection cancer has about 1 cm^3^ volume and contains 10^9^ cells. The average time of the preclinical phase lasts from 15 to 20 years, or much longer, even 50 years. This raises the question of whether the mutations present in the mitochondrial DNA are primary in relation to cancer or are the result of changes produced and processes occurring during carcinogenesis. Their original character is indicated by their disclosure in the form of clinical signs after a long period of occurrence (upon reaching the prevalence of the mutant mtDNA) and the slow progressive nature of the symptoms as is the case with the mitochondrial diseases.
